# Age at menarche and risk of multiple sclerosis (MS): a systematic review and meta-analysis

**DOI:** 10.1186/s12883-019-1473-5

**Published:** 2019-11-14

**Authors:** Amirreza Azimi, Sara Hanaei, Mohammad Ali Sahraian, Mehdi Mohammadifar, Sreeram V. Ramagopalan, Mahsa Ghajarzadeh

**Affiliations:** 1 Multiple Sclerosis Research Center, Neuroscience Institute, Tehran University of Medical Sciences, Tehran, Iran; 2 Research Center for Immunodeficiencies (RCID), Tehran University of Medical Sciences (TUMS), Tehran, Iran; 3 Universal Scientific Education and Research Network (USERN),Tehran university of medical sciences, Tehran, Iran; 4 Department of Radiology, Zanjan University of Medical Sciences, Zanjan, Iran; 5 Bristol-Myers Squibb, Uxbridge, UK; 6 Universal Council of Epidemiology (UCE), Universal Scientific Education and Research Network (USERN), Tehran University of Medical Sciences, Tehran, Iran

**Keywords:** Menarche, Multiple sclerosis, Risk

## Abstract

**Background:**

Some studies have looked at the age at menarche and risk of Multiple Sclerosis (MS).We aimed to conduct a systematic review and meta-analysis to estimate a pooled odds ratio of developing MS by increasing age at menarche.

**Methods:**

We searched PubMed, Scopus, EMBASE, CINAHL, Web of Science, Ovid, google scholar and gray literature (references of references, congress abstracts) up to 10th April 2019.

**Results:**

The literature search found 312 articles. After eliminating duplicates, reviews, case reports and trials, 18 articles remained. Three articles were ultimately included in the final analysis. Two studies were from Iran, and one from Canada. The pooled odds ratio (OR) for increasing 1 year of age at menarche was 0.88 (95% CI:0.82-0.94), with no significant heterogeneity (I^2^ = 49%, *p* = 0.1). Mean age at menarche was significantly different between case and control groups (mean difference = − 0.22, 95% CI = -0.42,-0.02).

**Conclusion:**

The result of this systematic review showed that the risk of MS decreases by increasing age at menarche.

## Background

Multiple sclerosis (MS) is an autoimmune disease affecting women more than men and is the most frequent leading cause of neurological disability in young adults along with trauma [1–3]. Different factors including genetics, as well as environmental factors such as smoking, Epstein-Barr virus infection, latitude of residence, and vitamin D status, have been considered as associated risk factors of MS [4, 5].

Although MS appears mostly in young adults, pediatric MS is now prevalent and there are challenging issues regarding its occurrence [6].

Previous studies have shown that earlier menarche is associated with an increased risk of various diseases such as breast cancer and type 2 diabetes [7, 8].

In women, sex hormones have crucial roles in the immune system development which leads to higher levels of immunoglobulins, strong activation of T-cell and more antibody response reactions to antigens [9]. Previous case-control studies demonstrated that age at menarche is lower in women with MS than healthy controls however, the magnitude of the effect of this association differs between studies [10, 11].

In a recent case-control study conducted in Iran, Salehi et reported 8% reduction of MS risk for each one-year increase of menarche age [12].

As the age of menarche differs in different countries and published articles reporting odds of MS by increasing age at menarche, we aimed to conduct this systematic review and meta-analysis to estimate a pooled odds ratio of developing MS by increasing age at menarche.

## Methods

### Literature search

We searched PubMed, Scopus, EMBASE, CINAHL, Web of Science, Ovid, Google scholar and Gray literature (references of references, congress abstracts) up to 10th April 2019.

Inclusion criteria were:

1. Case-control studies
2. Studies providing crude odds ratio (OR) for the age of menarche and risk of MS
3. Articles published in the English language

### Data search and extraction

The search syntax for identifying studies was:

(Puberty OR menarche) AND (Multiple Sclerosis OR Sclerosis, Multiple) OR Sclerosis, Disseminated) OR Disseminated Sclerosis) OR MS (Multiple Sclerosis)) OR Multiple Sclerosis, Acute Fulminating).

Data extraction and evaluation of studies were performed by two independent researchers. Name of the first authors, publication year, country, number of cases in each group of the study, crude OR, lower limit and upper limit of 95% CI of crude ORs were extracted.

### Risk of bias assessment

The risk of bias was assessed by the modified NEWCASTLE - OTTAWA QUALITY ASSESSMENT SCALE (for case-control studies) [13] (Additional file 1).

### Statistical analysis

STATA Version 13.0 (Stata Corp LP, College Station, TX, USA) and RevMan 5.3 (The Cochrane Community, London, United Kingdom) were used for data analysis. Random effects models were used and heterogeneity was determined by the inconsistency (I^2^) calculation. Accordingly, and as discussed by Deeks et al. [14] before, the I^2^ of more than 40% was considered high for heterogeneity. Mean difference was calculated for the age at menarche comparison.

## Results

**Fig. 1:**
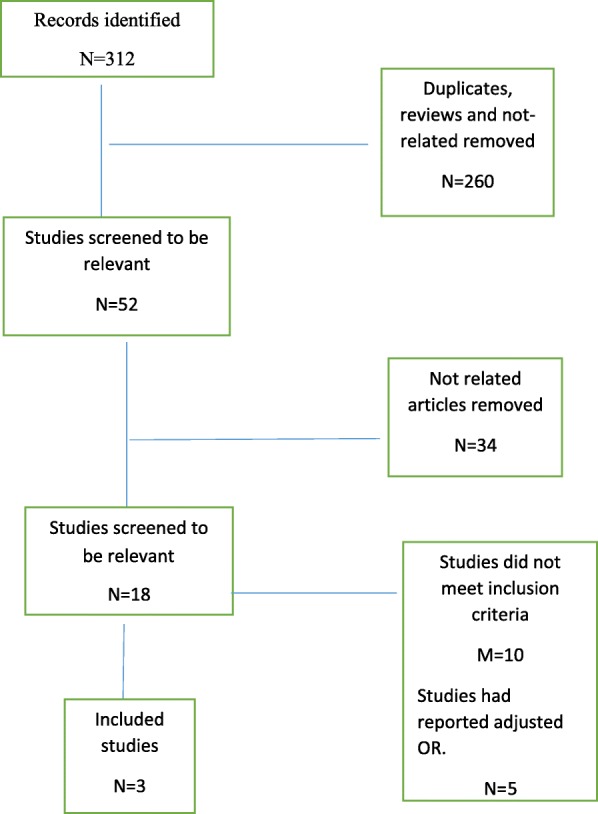
Flow diagram showing the selection of eligible studies

We found 312 articles in the first search and after eliminating duplicates, reviews and unrelated articles, 52 remained. Full-text evaluation led to the inclusion of 18 articles while only 3 remained for the meta-analysis (Fig. 1). Overall, 5071 cases and 1842 controls were analyzed.

Two studies were from Iran, one from Canada, and one from Denmark (Table 1).

**Table 1:** Characteristics of included studies

First author	Published year	Country	Type of study	No case/No control	OR(95% CI)
Ramagopalan [15]	2009	CANADA	case-control	4472/ 1101	0.89(0.83-0.94)
Salehi [12]	2018	Iran	case-control	399/541	0.92(0.84-0.99)
Rejali [16]	2016	Iran	case-control	200/ 200	0.78(67-0.89)

**Fig. 2:**
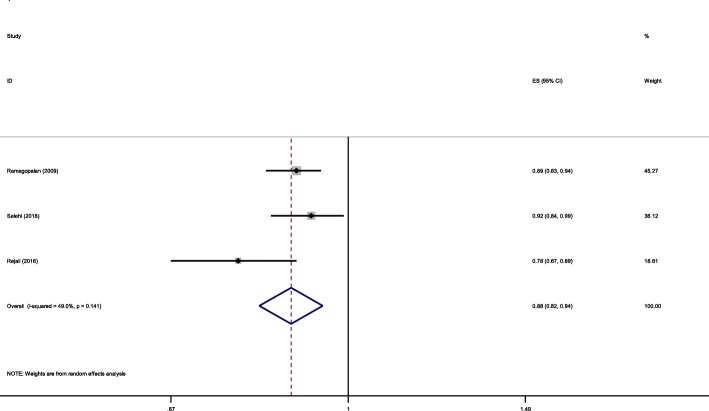
Forest plot showing pooled OR of age at menarche and risk of MS

OR for age at menarche and risk of MS differed between studies ranging from 0.78 to 0.92. The pooled OR for increasing 1 year of age at menarche was 0.88 (95% CI:0.82-0.94) (The CI do not include one) (I^2^ = 49%, *p* = 0.1) (Fig. 2).

This means that each 1 year increase of age at menarche will result in 12% reduction of MS odds.

**Fig. 3:**
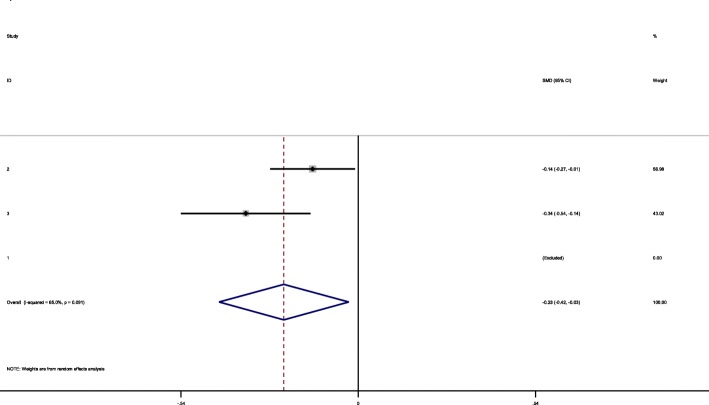
Mean age difference in MS and control groups

Except for Ramagopalan et al. study, all other studies provided mean age at menarche in case and control groups. Mean age at menarche was significantly different between case and control groups (mean difference = − 0.22, 95% CI = -0.42,-0.02) (The CI do not include zero) (Fig. 3).

All included studies had good quality (≥5) using NEWCASTLE - OTTAWA QUALITY ASSESSMENT SCALE (for case-control studies) (Table 2).

**Table 2:** quality assessment of case-control studies

First author	Case definition	Representativeness	Selection of controls	Definition of controls	Comparability	Ascertainment of exposure	Same method of ascertainment	Non-response rate	score
Salehi	a	a	a	a	a	c	a	b	6
Rejali	a	a	a	a	a	c	a	b	5
Ramagopalan	a	a	a	a	a	c	a	b	6

## Discussion

To our knowledge, this is the first systematic review and meta-analysis evaluating age at menarche and risk of MS.

The pooled OR for increasing 1 year of age at menarche was 0.88 (95% CI:0.82-0.94) (The CI do not include one) which shows that each year increase of age will result in 12% reduction of MS odds. The OR for age at menarche and risk of MS differed between studies ranging from 0.78 to 0.92. The difference in reported odds could be due to different sample sizes and patient characteristics.

Mean age at menarche was significantly different between case and control groups (mean difference = − 0.22, 95% CI = -0.42, − 0.02) (The CI do not include zero). There are controversies regarding the findings of previous studies about age at menarche in MS cases and controls. For the first time, Antonovsky et al. examined the age at menarche in MS female cases and controls and found no significant difference [17].

Operskalski et al. compared 108 MS women with 108 healthy controls and reported mean age at menarche in cases as 12.1 years vs 12.7 years in controls (*p* = 0.01) [11].

Gustavsen et al. compared 391 MS patients and 535 female controls and reported no significant difference (13.07 vs. 12.97) [18].

MS is a complex neurological disease and the exact causative factors are not yet conclusively determined. Female domination is prominent and the relationship between menarche age and risk of MS may demonstrate the role of sex hormones in MS development [19]. Estrogen has known effects on CNS development as well as the immune system [9]. Early menarche may result in an imbalance of estrogen and predispose women to MS development [15].

Although puberty affects brain development, brain development affects puberty conversely by activating GnRH neurons [20]. Sloka et al. reported the relationship between age at menarche and age of first MS symptoms presentation [19]. Their results showed that a 1 year increase in menarche age resulted in MS symptom appearance 1.16 years later [19]. Previous studies have also demonstrated a significant decrease of relapse rate during pregnancy and increase immediately after delivery [21, 22].

It has been suggested that elevated levels of estrogen during pregnancy results in relapse rate reduction and estradiol protects oligodendrocytes from death [23].

There are controversies regarding the use of contraceptives and the risk of MS. Salehi et al. found that use of oral contraceptive pill (OCPs) will increase risk of MS development by 40% [12] which is in agreement with D’hooghe et al. who found that OCPs increases the risk of disease progression in MS [24] while other studies found no relationship [25, 26]. It is possible gonadotropin secretion and ovulation inhibition causes CNS hormonal imbalance increasing the risk of MS following OCPs use [12, 26].

To our knowledge this is the first meta-analysis to assess age at menarche and risk of (MS) which has limitations. First the included studies do not cover female cases of all ethnicities.

Second, the number of cases and controls are not the same in all included studies. The difference between the case-control-ratio may cause bias.

Third, the number of included studies are limited.

On the other hand, I^2^ parameter was 49 and 65% in two meta-analyses of the current study, which was indicative for high heterogeneity level as defined by Deeks et al. [14].

Altogether, more studies including findings from patients of all five continents with larger sample sizes are recommended. Also, more precise results could be achieved in case the included studies are more homogenous, both clinically and statistically.

## Conclusion

The result of this systematic review showed that the risk of MS decreases by increasing age at menarche.

## Supplementary information

**Additional file 1:** MODIFIED NEWCASTLE - OTTAWA QUALITY ASSESSMENT SCALE for quality assessment for cohort and case-control studies.

## Data Availability

The search syntax for identifying studies was: (Puberty OR menarche) AND (Multiple Sclerosis OR Sclerosis, Multiple) OR Sclerosis, Disseminated) OR Disseminated Sclerosis) OR MS (Multiple Sclerosis)) OR Multiple Sclerosis, Acute Fulminating) which was searched in PubMed, Scopus, EMBASE, CINAHL, Web of Science, Ovid, Google scholar.

## Abbreviations

MS: Multiple Sclerosis
OR: Odds Ratio
