# Supplementary material for: Age at menarche and risk of multiple sclerosis (MS): a systematic review and meta-analysis
Source: BMC Neurol. 2019 Nov 14;19:286. doi: 10.1186/s12883-019-1473-5 (PMC6854684; doi:10.1186/s12883-019-1473-5)
Supplement: Supplementary file 1 — Additional file 1: MODIFIED NEWCASTLE - OTTAWA QUALITY ASSESSMENT SCALE for quality assessment for cohort and case-control studies. [file 12883_2019_1473_MOESM1_ESM.pdf]

MODIFIED NEWCASTLE - OTTAWA QUALITY ASSESSMENT SCALE

CASE-CONTROL STUDIES

Note: A study can be awarded a maximum of one star for each numbered item within the Selection and Exposure categories. A maximum of two stars can be given for Comparability.

No.	Criterion	Decision rule	Score: (*=1 no*=0)	Location in text
**SELECTION**				
1	Is the case definition adequate?	a) Yes, with independent validation (>1 person/record/time/process to extract information, or reference to primary record source such as x-rays or structured injury data)*<br>b) Yes, based on self-reports<br>c) No description		
2	Representativeness of the cases	a) All eligible cases with outcome of interest over a defined period of time, all cases in a defined catchment area, all cases in a defined team/competition/sport, or a random sample of those cases*<br>b) Not satisfying requirements in part (a), or not stated.		
3	Selection of controls	a) Controls were selected from the same source population as the cases*<br>b) controls were selected from a different source population<br>c) no description		
4	Definition of controls	a) If cases are first occurrence of injury of interest, then it must explicitly state that controls have no history of this outcome. If cases have new (not necessarily first) occurrence of specific injury, then controls with previous occurrences of outcome of interest should not be excluded*<br>b) No description of injury history		
**COMPARABILITY**				
1	Comparability of cases and controls on the basis of the design or analysis	a) Study controls for previous injury*<br>b) Study controls for age*<br><br>*Note:* Cases and controls must be matched in the design and/or confounders must be adjusted for in the analysis. Alone statements of no differences between groups or that differences were not statistically significant are not sufficient.		
**EXPOSURE**				
1	Ascertainment of exposure	a) Structured injury data (e.g. record completed by medical staff)*<br>b) Structured interview where blinded to case/control status*<br>c) Interview not blinded to case/control status<br>d) Written self-report or medical record (unstructured data) only<br>e) No description		
2	Same method of ascertainment for cases and controls	a) Yes*<br>b) No		
3	Non-response rate	a) Same for both groups*<br>b) Non-respondents described<br>c) Rate different and no designation		
		**SCORE:**		

MODIFIED NEWCASTLE - OTTAWA QUALITY ASSESSMENT SCALE

COHORT STUDIES

Note: A study can be awarded a maximum of one star for each numbered item within the Selection and Outcome categories. A maximum of two stars can be given for Comparability

**No.**	**Criterion**	**Decision rule**	**Score (*=1, no*=0)**	**Location in text**
**SELECTION**				
1	Representativeness of the exposed cohort	a) Consecutive eligible participants were selected, participants were randomly selected, or all participants were invited to participate from the source population*<br>b) Not satisfying requirements in part (a), or not stated.		
2	Selection of the non-exposed cohort	a) Selected from the same source population*<br>b) Selected from a different source population<br>c) No description		
3	Ascertainment of exposure	a) Structured injury data (e.g. record completed by medical staff)*<br>b) Structured interview*<br>c) Written self-report<br>d) No description		
4	Demonstration that outcome of interest was not present at the start of the study	a) Yes*<br>b) No or not explicitly stated		
**COMPARABILITY**				
1	Comparability of cohorts on the basis of the design or analysis	a) Study controls for previous injury*<br>b) Study controls for age*<br><br>*Note:* Exposed and non-exposed individuals must be matched in the design and/or confounders must be adjusted for in the analysis. Alone statements of no differences between groups or that differences were not statistically significant are not sufficient.		
**OUTCOME**				
1	Assessment of outcome	a) Independent or blind assessment stated, or confirmation of the outcome by reference to secure records (e.g. imaging, structured injury data, etc.)*<br>b) record linkage (e.g. identified through ICD codes on database records)*<br>c) Self-report with no reference to original structured injury data or imaging<br>d) No description		
2	Was follow-up long enough for outcomes to occur?	a) Yes (≥3 months)*<br>b) No (<3 months)		
3	Adequacy of follow up of cohorts	a) Complete follow up – all participants accounted for*<br>b) Subjects lost to follow up unlikely to introduce bias (<15% lost to follow up, or description provided of those lost*)<br>c) Follow up rate <85% and no description of those lost provided<br>d) No statement		
		**SCORE:**		
